# Inflammatory Malignant Fibrous Histiocytoma Associated with Leukemoid Reaction or Leukocytosis: A Comprehensive Review

**DOI:** 10.5402/2012/946019

**Published:** 2012-10-14

**Authors:** Jorge Hurtado-Cordovi, Prajwol Pathak, Boris Avezbakiyev, Marianne Frieri

**Affiliations:** Divisions of Hematology and Oncology and Allergy and Immunology, Department of Medicine, Nassau University Medical Center and North Shore- Long Island Jewish Health Care System, 2201 Hempstead Turnpike, East Meadow, NY 11554, USA

## Abstract

Inflammatory malignant fibrous histiocytoma (IMFH) associated with leukemoid reaction (LR)/leukocytosis is a rare entity. In this paper, we search PubMed for all known cases of IMFH associated with LR/leukocytosis in an attempt to draw conclusions about this variant's response to treatments and its pathophysiology. Medline electronic database was searched using key words such as malignant fibrous histiocytoma, leukemoid reaction, and leukocytosis. A total of 16 patients were found, twelve males (75%) and 4 female (25%), with a mean age of 62.6 years, ranging from 47 to 77. The mean survival was 770 days, ranging from 14 to 6570 days. Four patients were alive at last follow-up: 6570 days, 1095 days, 335 days, and 180 days, respectively. Of the 12 patients that expired, death occurred approximately 92 days after the onset of LR or leukocytosis, ranging from 3 to 334 days. We conclude that IMFH associated with LR/leukocytosis does not completely respond to chemoradiation. Overproduction of growth factors and cytokines by IMFH cells and their interactions with the inflammatory infiltrate seem to promote immunological effector cell's dysfunction and substantiate the development and growth of this neoplasm. A clear understanding of these molecular pathways is crucial in order to identify targets for potential therapy.

## 1. Introduction

Inflammatory malignant fibrous histiocytoma (IMFH), also known as undifferentiated pleomorphic sarcoma (UPS) inflammatory variant, was initially identified as a separate category by Kyriakos and Kempson in 1976 [1]. Its name is derived from its distinct histological pattern; an intense inflammatory infiltrate that may be composed of neutrophils, eosinophils, and/or lymphocytes without a recognized source of infection. These tumors are usually bulky, and their clinical course is characterized by multiple local recurrences, metastasis, and ultimately death. Due to its mesenchymal origins, this neoplasm can virtually affect any part of the body; however, they are most commonly encountered in the retroperitoneal cavity. Patients typically present with fever and other constitutional symptoms mimicking an infectious process, and/or a rapidly growing painful mass. In cases of retroperitoneal IMFH, patients can also present with additional signs/symptoms of a space-occupying lesion [2–6].

Conventionally, leukemoid reaction (LR) is defined as a secondary leukocytosis with a white blood cell count (WBC) that is frequently above 50 K/mm^3^ resulting in a severe left shift with the presence of immature myeloid cells in the peripheral blood, but with clear distinction of polyclonality and no signs of maturation arrest. Although it resembles leukemia, the presence of this disease cannot be demonstrated during the course of the illness or postmortem. Nowadays experts agree that the WBC count does not necessarily have to exceed 50 K/mm^3^  to be considered a LR as long as the parameters described above are met [7, 8]. IMFH is universally associated with an elevated white blood cell count (WBC) composed mostly of neutrophils [9, 10]. Due to the severe elevation in the WBC, some of these cases have been misdiagnosed as acute leukemias; however, bone marrow biopsy, when performed, typically shows hypercellularity without evidence of monoclonal proliferation [1, 9, 11]. A few reports have documented a normalization of the WBC count after removal of the primary neoplasm, and an increase to previous values or higher once the tumor relapses [12–17]. This finding suggests that IMFH is capable of releasing a variety of cytokines and growth factors which could contribute to the development of the LR/leukocytosis associated with it [9, 10, 12, 15, 16, 18, 19]. 

The causes of the LR associated with this malignancy are not well understood since there are so few articles reported in the primary medical literature. In this paper, we search PubMed for all known cases of IMFH associated with LR or leukocytosis in an attempt to draw conclusions regarding this variant's clinical presentation, course, survival, available treatments, and the pathophysiology behind the LR/leukocytosis.

## 2. Methods

 Medline (PubMed) electronic database was searched by two independent reviewers (JH and PP) using key words such as “malignant fibrous histiocytoma, leukemoid reaction, pleomorphic undifferentiated sarcoma inflammatory variant, and leukocytosis.” Older nomenclatures for this tumor such as “xanthosarcoma, malignant fibrous xanthoma, and inflammatory fibrous histiocytoma” were included in our search as well. Other types of sarcomas and undifferentiated Sarcomas associated with leukemoid reaction or leukocytosis were excluded. Leukemoid reaction was defined according to the parameters described in the Introduction section. Leukocytosis was defined as a WBC two standard deviations above the mean with the predominance of a specific mature myeloid cell, lymphocyte, or monocyte. If the WBC was less than 50 K/mm^3^, but a severe left shift was present, early myeloid cells wereseen in circulation, polyclonality was identified, and there were no signs of maturation arrest; then it was considered as a leukemoid reaction. The results were screened according to but not limited to the following exclusion criterion: patient younger than 18, iatrogenic-related tumors (such as postradiation), tumor arising secondary to preexisting disease, synchronous or metachronous malignancies, and IMFH associated with a maximum WBC less than 24 K/mm^3^. 

## 3. Results

 After a detailed search of Medline, according to the exclusion criterion described in the Methods section, we found 14 articles. One of the papers was a primary research article (Melhan et al.), one a case series (Kyriakos et al.), one a brief report (Algabra et al.), and the rest were case reports (Table 1). A total of 16 patients were described by these studies, 12 (75%) males and 4 (25%) females (Table 2). The mean age at presentation was 62.6 years with a range of 47–77. A total of 8 tumors were located in the retroperitoneal space (50%), 2 of them in solid organs outside the peritoneal cavity, 2 were found inside the abdominal cavity, 3 were located in the extremities/deep soft tissue, and 1 was confined to the dermis (Table 2). The most common presenting complaints were a combination of mass/pain in 13 patients (81.2%) and constitutional symptoms in 7 patients (43.8%) with weight lost and fever being the most common. The mean survival was 770 days, ranging from 14 to 6570 days. Four patients were alive at last followup: 6570 days, 1095 days, 335 days, and 180 days. Of those 12 patients that passed away, death occurred approximately 92 days after the onset of LR or leukocytosis, ranging from 3 to 334 days (Table 2).

## 4. Discussion

Inflammatory malignant fibrous histiocytoma*∖*undifferentiated pleomorphic sarcoma inflammatory variant was first described over 30 years ago; however, due to the rarity of this entity, information about its presentation, clinical course, and outcome is very scarce. Unfortunately, our analysis is greatly limited by the small number of cases found in the primary medical literature. Taking this limitation into account, we attempt to draw conclusions regarding the clinical behavior of this neoplasm. Our results suggest that IMFH associated with LR/leukocytosis usually presents in the seventh decade, and up to 75% of the cases are seen in males. Thirteen patients (81.2%) presented complaining of a combination of a mass and/or pain. Forty percent of these individuals complained of constitutional symptoms, weight lost and fever being the most frequent. Our study suggests that 50% of this variant arises from the retroperitoneal space. However, due to its mesenchymal origin, it is known that this neoplasm can virtually affect any organ [20, 21]. The previous statement correlates with some of the cases described in which IMFH with/without LR has been detected in solid organs, intestinal tract, musculoskeletal tissue, and the integument [12, 14, 17, 19, 22, 23].

The overall survival observed varies greatly from case to case. Perhaps this finding portrays the different treatments, locations, and how advanced the disease was at presentation. The mean overall survival was 770 days with a range of 14–6570 days; four patients were alive at last followup (Table 2). Twelve patients passed away; on average, death occurred approximately 92 days after the onset of LR or leukocytosis. Our analysis shows that the severity of the leukemoid reaction correlates with the time of death. Close to the time of demise, in both of the cases reported by Melhan et al., the WBC increased above 150 K/mm^3^. Although, patient number 2 in this study survived for more than 400 days; once his WBC increased to a 3 digit value, he quickly deteriorated and expired [9]. We reported a similar case of a 60-year-old white male that presented with a severe leukemoid reaction; his clinical course was highlighted by rapid deterioration and death occurring secondary to cardiovascular arrest. It is worth noticing that in both of these studies once the WBC went above 100 K/mm^3^, the patients were not capable of tolerating any chemoradiation secondary to their unstable clinical condition. This observation reemphasizes the need for rapid detection and aggressive treatment in patients with this disease [1, 24–26]. 

On the other hand, of those 4 patients that were alive at last followup, it was observed that their WBC never increased above 50 K/mm^3^. Asirwatham and Pickeren reported a case that survived for 18 years (Table 1). The patient's tumor measured 10 × 7 × 4 cm and was located in the left thigh involving the muscles. The highest WBC registered was 38 K/mm^3^. Patient treatment was delayed due to 4 none diagnostic biopsies; finally, the patient received an excisional biopsy which confirmed the diagnosis of IMFH associated with LR. Patient had radiation treatment which was complicated with erosion of the femoral artery resulting in massive hemorrhage necessitating emergent left thigh amputation. The surgical specimen was positive for metastatic foci in the deep soft tissue despite negative surgical margins and two tumor-free saphenous lymph nodes. These findings suggest that this tumor does not completely respond to radiotherapy, and that perhaps limb amputation results in long-term survival [13]. However, the morbidity and psychological impact associated with this procedure have to be considered especially since in this case, the surgery was done due to treatment complications and not with a curative intent. Kato et al. also reported a case in which the patient survived for 3 years after original diagnosis. The clinical course of this patient was characterized by multiple recurrences and aggressive surgical intervention each time. The highest WBC was 50.4 K/mm^3^, and he was able to recover successfully from each surgical intervention [13]. It should be mentioned, that these two patients had no history of chronic debilitating diseases and had good functional statuses at disease presentation. Thus, these factors may have also played a role in the response to treatment. 

It also seems that the location of the tumor has an impact on survival and response to treatment. Algabra et al. reported a case of IMFH associated with LR located in the right epididymis. Patient presented with pain, fever, and the highest WBC was 32.5 K/mm^3^. Patient was treated aggressively with surgical removal of the right testicle followed by 10 cycles of chemotherapy (unfortunately the agents and doses were not specified), and local radiation with Cobalt^60^. Patient was alive and disease-free at last followup (Table 1) [17]. There were 8 patients diagnosed with retroperitoneal neoplasm of which 7 passed away, and only one survived. It is worth noting that the individual that survived was followed only for 180 days, which we considered to be a short period of time to label someone disease-free. This neoplasm has a high rate of local recurrence and metastasis, thus it is prudent to have a longer followup [14, 25]. These observations suggest that when this malignancy arises from the retroperitoneum it has a higher recurrence rate and a poorer prognosis. 

Our findings challenge the current view that depth of invasion at diagnosis correlates with metastatic potential [2, 16]. We report a case of cutaneous IMFH associated with LR which at presentation was restricted to the dermis, had disease-free surgical margins, and a chest X-ray that was negative for any metastasis. However, the patient expired approximately 38 days after surgery with wide spread pulmonary metastasis [26]. This tumor also has a high recurrence rate, as portrayed by the case described by Takahashi et al. This patient had multiple recurrences and surgery before he finally succumbed to the disease approximately 35 days after the onset LR. Finally, it was also noted that in most cases, the severe leukemoid reaction was a sign of advanced disease and suggestive of a very poor prognosis. On the other hand, resolution of the LR/leukocytosis and normalization of inflammatory makers were associated with remission; once this malignancy relapses, these parameters increase once more [9, 12–17].

Treatment for this neoplasm remains mostly surgical; however, this approach is often precluded due to the age of the patients, advance stage of the disease at presentation, and comorbidities [1, 2, 9, 25, 27, 28]. This tumor is not completely radiosensitive since relapses with wide spread metastasis have been observed after full courses of radiation, and deep soft tissue metastatic microfoci have been detected in surgical specimens after completion of radiation treatment [1, 9, 13]. The role of chemotherapy remains controversial; in our study, we found no clinical benefit or increase in overall survival with use of chemotherapy for metastatic and/or unresectable disease. However, it should be mentioned that in many of the cases described in this paper (Table 1) the fragile clinical condition of the patients precluded the use of intense chemotherapeutic plans. There have been reports of complete remission after the use of cyclophosphamide base chemotherapy in patients with IMFH not associated with LR. Poon et al. reported complete response to chemotherapy in four cases. The regimen consisted of cyclophosphamide in all cases, with additional doxorubicin, and methotrexate in the 1st case, with vincristine, procarbazine, and prednisone in the 2nd case, with doxorubicin, vincristine and prednisone in the 3rd, and with additional vincristine, and doxorubicin in the 4th case. They further suggested that inflammatory variant of malignant fibrous histiocytoma could be responsive to chemotherapy [25, 29, 30]. Phase II randomized trials conducted by The Sarcoma Alliance for Research Through Collaboration have shown that the combination of gemcitabine and docetaxel produced a 36% response rate (4 out 11 patients); this combination could be used for those patients refractory to cyclophosphamide/ifosfamide-based therapy. Unfortunately, this treatment was associated with significant pulmonary toxicity and refractory peripheral edema [31]. Early diagnosis of IMFH, preferably before the WBC reaches values above 50 K/mm^3^, is crucial in order to entertain the possibility of aggressive chemotherapy with curative intent. 

 The exact reason responsible for the development of the LR/leukocytosis seen with this neoplasm remains unclear. However, it is now widely accepted that IMFH arises from a mesenchymal cell progenitor [20, 21]. The predominant malignant cells seen in IMFH are primitive macrophages and/or histiocytes that retain the ability of cell division and phagocytosis. They can also release a variety of cytokines which correlate with their origin [9]. High levels of granulocyte colony-stimulating factor (G-CSF) have been detected in the serum of patients with IMFH associated with LR/leukocytosis (Table 1), as well as in other histological variants of this disease [12, 16, 18, 32, 33]. The role of G-CSF in the growth and progression of tumors remains controversial. Under normal homeostasis, it promotes the development of neutrophils which are a key component of the innate immune system. However, when G-CSF is overproduced by tumor cells, it has a tendency to encourage the development of what has been called myeloid-derived suppressor cells (MDSC) [34–39]. These immature immune cells are recruited from the bone marrow. They travel to the lymph nodes where dendritic cells prime T lymphocytes to mediate tumor cell elimination and hinder this process. The exact mechanism of how this is accomplished is still unknown; however, animal tumor models have shown that MDSC produce great quantities of reactive oxygen species (ROS) and reactive nitrogen species such as Peroxynitrite. ROS prevent maturation of dendritic cells while promoting additional accumulation of MDSC, perpetrating further immune dysfunction. Peroxynitrite can diffuse across cells membranes and are capable of producing posttranslational modifications by nitration of amino acid residues such as tyrosine, cysteine, methionine, and tryptophan. These modifications affect the tridimensional structure of T-Cell receptors (TCR). This in turn decreases the binding affinity of cytotoxic CD8^+^'s TCR for MCH I peptide complex, weakening their ability to respond to antigen challenges [40–42].

Animal models have also revealed that MDSC can also travel to tumors sites where they interfere with normal functioning of immune cells via the production of arginase 1. This enzyme rapidly depletes arginine from the tumor microenvironment which produces a severe inhibition of T-cell proliferation, diminish the expression of the CD3 zeta chain which is a key component of the TCR, and decreases the production of cytokines which promote tumor recognition and elimination. Simultaneous expression of arginase I and nitric oxide synthase (iNOS) by MDSC further enables them to restrain T-cell antitumor activity by stimulating the expression of these two pathways in nearby cells. They can also promote T-cell apoptosis by means of increasing nitric oxide concentrations. MDSC are also found in the blood of cancer patients, and elevated activity of these two enzymes has been observed as well. *In vitro* studies have shown that depletion of these cells from the circulatory system of these patients restores normal function of peripheral lymphocytes. Lastly, MDSC also produce and stimulate the production of cytokines such as IL 4 and IL 13 among others. These molecules promote tumor escape by encouraging the differentiation of T suppressor lymphocytes from precursor cells. In turn, T suppressor lymphocytes induce immunotolerance and anergia in tumor infiltrating lymphocytes. These observations suggest that MDSC have an active role in promoting immune cell dysfunction in the tumor microenvironment as well as in the peripheral circulation of cancer patients [41–43]. Other inflammatory cytokines such as IL-6, IL-7, IL-8, transforming growth factor beta (TGF-beta), and stem colony-stimulating factor (SCF), are present at abnormally high levels in the serum of patients with this neoplasm. Animal models have shown that IL-6 can sustain neutrophil and macrophage colonies, and through its action on other immune cells, it can stimulate the bone marrow to produce other growth factors. Interlukin-7 induces proliferation of lymphocytes; it also increases WBC by stimulating secretion of IL-1, IL-6, and tumor necrosis (TNF) factor from peripheral lymphocytes, monocytes, and macrophages. Interlukin-8 is a potent neutrophilic chemotactic and activating cytokine; thus it may contribute to the severe inflammatory infiltrate seen in IMFH lesions [9, 44, 45].

Under normal physiological conditions, TGF-beta controls key cell functions such as proliferation, differentiation, and it also promotes extracellular matrix formation and tissue repair. Generally speaking, TGF-beta inhibits proliferation of epithelial cells, and it encourages growth of some mesenchymal cell subsets; however, its concentration as well as the density of its receptors has been found to be overexpressed in many solid malignancies. This observation has lead researchers to believe that it may play a central role in the development and growth of tumors. Yamamoto et al. investigated the expression of TGF-beta isoforms and their receptors in 43 human MFH specimens. In this study, all the samples expressed different isoforms of this growth factor. On the other hand, 84 percent of these cells expressed TGF beta receptor type 1 (R1), 56% were positive for TGF beta R2, and 53% of them stained for both. These researchers compared the growth rate of MFH cells positive for both receptors with those positive only for one. They found that the MIB-1 proliferation index in the MFH cell line positive for both receptors was considerably higher than in those cells positive for a single one. This result suggests that the interactions of these receptors with their ligands have an important role in supporting the proliferation of MFH cells likely through an autocrine and/or paracrine mechanism. Thus, further description of the TGB R1 and R2 coexpression may aid oncologists in predicting the behavior of this tumor [46, 47]. Thus, we conclude that the high concentration of G-CSF, TGF-beta, inflammatory cytokines, and the abundant inflammatory infiltrate associated with this malignancy does not necessarily translate into an effective immune response; instead, these biomolecules and immature immune cells may be promoting the growth and aggressive nature of this neoplasm.

 SCF along with other grow factors act on the BM to increase the number of circulating erythrocytes, neutrophils, lymphocytes, monocytes, eosinophils, and basophils; it also contributes to the hypercellular BM observed in patients with this illness. The soluble and membrane-bound forms of SCF messenger RNAs have been detected in MFH cell lines. Membrane-bound SCF increases the stability of its receptor tyrosine-protein kinase Kit (c-Kit), thus avoiding c-Kit-down regulation and promoting longer Kinase activity. In addition, this membrane-bound form facilitates cell-to-cell adhesion by interacting with its receptor kinase, and since it has a cellular domain, it could also be capable of signal transduction either by itself or in conjunction with other biomolecules. Its soluble counterpart fulfills similar functions but is less potent. Based on these observations, researchers have theorized that the interactions between this growth factor with its receptor may sustain the development of these malignant cells likely through a paracrine mechanism [48, 49]. 

Other investigators disagree with this theory since they have found very low concentrations of c-Kit in MFH cell lines [50]. Under normal circumstances, SCF promotes the differentiation and development of mast cells and also induces the production of platelet-derived growth factor (PDGF), basic fibroblastic grow factor, vascular endothelial growth factor, among others by these cells. It is also known that c-Kit is structurally and functionally related to the PDGF receptor. It may be possible that increased manufacturing of SCF by these malignant cells could promote the production of these angiogenic substances which indirectly will promote the development and the metastatic potential of this neoplasm. This statement is supported by Taniuchi et al. which suggested that proliferation of these malignant cells could be mediated via an autocrine or paracrine pathway through PDGF receptors [51, 52]. In addition, animal studies have shown that tumor infiltrating MDSC along with other immature immune cells which are also part of the tumor's microenvironment can increase the bioavailability of PDGF. This growth factor has powerful angiogenic properties and promotes tumor growth and neovascularization through expression of metalloproteinase 9 by myeloid cells. Thus, SCF through direct and/or indirect actions could be one of the main culprits responsible for aggressive behavior display by this malignancy [53].

We speculated that since SCF and PDGF-beta seem to encourage the growth of this tumor through interactions with receptors on tumor cells and/or bystander cells; the thought of blocking of these receptors with multiselective kinase inhibitors such as imatinib or sunitinib could be entertained. These drugs can inhibit PDGF receptor kinase, Abl kinase, and the c-Kit receptor. Thus, they could theoretically prevent the interaction of these growth factors with their target and serve as an adjuvant therapy. This theory is supported by Irsan et al.'s results. These researchers demonstrated the expression of PDGF and c-Kit mRNA in 3 of their 4 MFH cell lines. These cells were cultured and transplanted into mice, thus creating a Xenograft model. Imatinib was found to inhibit tumor growth in those MFH cells that were positive for PDGF and c-Kit receptor, but not in those that were negative. This group concluded that imatinib may work by preventing phosphorylation at the kinase receptor domain. On the other hand, sunitinib in particular has shown activity against other solid tumors, even in cases of gastrointestinal stromal tumors with documented resistance to imatinib. Mahmood et al. conducted a single institution phase II trial to investigate the safety and efficacy of this Kinase inhibitor in 3 types of sarcomas including MFH. The patients in this trial had metastatic or unresectable disease. This team found a median progression-free survival and overall free survival of 4.2 and 13.6 months, respectively. Unfortunately, their data failed to reach any statistical significance, and they could not show any significant increase in overall response. However, there were confounders in this research that could have affected the outcome; namely, a heavily pretreated population and patients with indolent malignancy. Thus, we believe that further study of these agents with a larger sample size and in a randomized pattern is warranted [54, 55].

IMFH associated with LR carries an overall poor prognosis. The presence of LR occurs once there is advanced disease, and it suggests a dismal prognosis. The tumor does not completely respond to XRT and we did not observe any clinical benefits whenever chemotherapy was used likely secondary to the brittle clinical condition of the patients. Further studies of multiselective kinase inhibitors such as imatinib and sunitinib are warranted since they have shown some activity against this malignancy; however, they should be done in bigger sample size population and in a randomized pattern. Early detection and accurate diagnosis is crucial in order to attempt chemotherapy with curative intents. Overproduction of growth factors and cytokines by IMFH cells and their interactions with the inflammatory infiltrate seem to promote immunological effector cell's dysfunction and substantiate the development and growth of this neoplasm. Thus, more research is needed to understand the exact mechanism of how these substances favor the progression of these neoplastic cells. This will eventually lead to the identification of molecular targets for treatment. 

## Figures and Tables

**Table 1:** Shows the characteristics and relevant aspects of the patients with IMFH associated with LR or leukocytosis.

Study	Patient characteristics	Tumor characteristics	WBC (highest)	Treatment	Survival	Comments
Hisaoka et al. *Pathology international* (1997) Case report	Sex: male (on hemodialysis) Age: 69 Patient presented c/o dull pain in the left loin and thigh for 1 month; no constitutional complaints.	Tumor located within the left Iliopsoas muscle 2.5 cm in diameter; originally misdiagnosed as an intramuscular abscess. No metastasis.	WBC: 73.9 K/mm^3^ range ( 18–73.9) (no differential reported) After the first surgery WBC returned to normal and it increased again once tumor recurred.	Surgery done twice due to local recurrence. Patient received chemotherapy after the second surgery (details not reported.)	Death: Approx. 180 days after diagnosis due to severe emaciation and liver dysfunction. Patient passed away approx. 150 days after the onset of leukemoid reaction.	This group suggested that retroperitoneal IMFHs could originate from dediffenciated Liposarcomas. Elevated serum concentration of G-CSF = 109.0 pg/mL, IL-6 = 95.8 pg/mL were seen after the first surgery due to tumor relapse, and they declined after the second one.

Melhem et al. *Blood* (1993) Primary article	Patient no. 1 Sex: male Age: 63 Patient presented with 1 year history of a growing abdominal mass and weight lost. Patient no. 2 Sex: male Age: 67 Patient presented with anorexia, fatigue, and a 10 kg weight loss for the past month	Patient no. 1: Tumor located in the retroperitoneum from L (lumbar) 3 to posterior mediastinum, 11 × 22 cm in diameter. No metastasis. Patient no. 2: Tumor located in the retroperitoneum involving the right kidney with wide spread metastasis to the liver and the omentum (dimensions not specified.)	Patient no. 1: WBC 164 K/mm^3^ range (58–164) Bands N: 75.44 K/mm^3^ Eos: 39.36 K/mm^3^ Meta: 16.4 K/mm3 Myel: 9.84 K/mm^3^ Promy: 8.2 K/mm^3^ Blast: 1.64 K/mm^3^ Patient no. 2: WBC 156 K/mm^3^ range (62–156) Bands N: 106.8 K/mm^3^ Neut: 43.68 K/mm^3^ Meta: 3.12 K/mm^3^	Patient no. 1: Partial resection with 5–10 % of tumor left. Radiation therapy given (details not reported.) Re-admitted 1 year after with increase residual mass. Patient no. 2: Patient deteriorated and emergency laparotomy was performed with finding as described in the tumor characteristics section. No other treatment was done secondary to patient's rapid demise.	Patient no. 1 Death: Approx. 489 days after diagnosis. Patient passed away about 180 days after the onset of leukemoid reaction. Immediate cause of death not reported. Patient no. 2 Death 14 days after diagnosis. Patient passed away 14 days after the onset of leukemoid reaction. Immediate cause of death not reported.	BM biopsy of both patients showed hypercellularity, no cytogenetic abnormalities, and no evidence of hematological malignancy. Alkaline phosphatase score was elevated in both patients. IL-6, IL-7, IL-8, TGF-beta, SCF, and KGF were detected in IMFH tumors with leukemoid reaction only. Researchers concluded that these cytokines could promote the growth and metastasis of this malignancy.

Kyriakos and Kempson *Cancer* (1976) Case series	Patient no. 1 (case 2) Sex: female Age: 47 Patient presented with a left lower quadrant mass that has been progressively growing, and subsequently became painful; no constitutional complaints. Patient no. 2 (case 6) Sex: Male Age: 60 Patient presented with weakness, night sweats, decrease exercise tolerance, and 22 lbs weight lost for 4 months. Fever develops shortly after disease onset.	Patient no. 1 Left lower quadrant abdominal mass with multiple recurrences. Patient was misdiagnosis several times including granulomatous inflammatory reaction unknown type, and pseudotumor inflammatory type. At autopsy a 3.5 × 4 × 2 cm mass was found on the mesentery of the small intestine, 100 cm distal to the ligament of Treitz with wide spread metastasis and bowel necrosis. Patient no. 2 Misdiagnosed with anemia, and then with leukemia before the diagnosis of neoplastic disease was entertained. Tumor found in the left upper quadrant measuring 8.5 cm in diameter adjacent to the left kidney. The neoplasm recurred in the right lower lung lobe, invading the chest wall, and extending into the retroperitoneum with wide spread metastasis and areas of necrosis.	Patient no. 1: WBC 30 K/mm^3^ (no other reported), no differential. Patient no. 2 WBC 86 K/mm^3^ range (17.1–86) Bands N: 63 K/mm^3^ Neut: 31 K/mm^3^ WBC normalized after first surgery and then increased once the tumor recurred.	Patient no. 1: A total of 4 surgeries done. First, an excisional biopsy reported as granulomatous inflammatory reaction undetermined type. Second was an exploratory laparotomy which showed an abdominal abscess, a dermoid cyst, and uterine myomas; no malignancy diagnosed at this time. Third surgery revealed an abdominal mass situated between the rectus muscle sheet and the peritoneum; it was diagnosed as inflammatory pseudotumor. Fourth surgery done due to recurrent of the abdominal mass. Patient received maximum dose of nitrogen mustard, and radiation therapy with1040 rads to the lower abdomen, and 632 rads to the upper which caused symptoms relieve. Patient no. 2 Surgical resection of the mass follow by radiation with 3800 rads to the tumor bed and para-aortic lymph nodes. Second surgery due to recurrence with right lower lung lobectomy along with preoperative radiation of 2025 rads; tumor partially removed. Tumor recurred and chemotherapy with adriamycin, dactinomycin, vincristine, and cyclophosphamide was attempted but failed.	Patient no. 1 Death Approx. 562 days after diagnosis. (Death occurred 114 days after leukocytosis onset.) Patient final days marked by neurological dysfunction associated with seizure, jaundice, low platelet and decrease WBC with a left shift, along with generalized weakness and cachexia. Immediate cause of death not reported. Patient no. 2 Death Approx. 2225 days after diagnosis. (Death occurred 36 days after leukemoid reaction onset.) Final clinical course characterized by persistent hypoglycemia, elevated alkaline phosphatase, oliguria, and pneumonia. Immediate cause of death not reported	Patient no. 1 At the onset of leukemoid reaction patient complained of fever, chills, and abdominal pain. During his last relapse, Patient was treated with Amphoteracin B because the mass was thought to be of infectious origin due to a positive serum complement fixation test for blastomycosis. BM aspiration showed myeloid hyperplasia. Thus, clinical course highlighted by multiple misdiagnoses which delayed treatment. Patient no. 2 At presentation misdiagnosed multiple times; fist with anemia and subsequently with leukemia. After the first surgery misdiagnosed as having Hodgkin's Lymphoma. BM biopsy showed granulocytic hyperplasia. Once more, patient's clinical course characterized by several misdiagnoses which delayed treatment.

Asirwatham et al. *Cancer* (1978) Case report	Sex: Male Age: 47 Patient presented with a 7 months history of left thigh pain which began after trauma, at first intermittent and then became progressively worse.	Tumor measured 10 × 7 × 4 cm and was located in the left thigh involving the muscles. After biopsy, diagnosis was not clear; nodular tenosynovitis, dermatofibroma, pigmented villonodular synovitis, atypical xanthogranuloma; among others, were some of the differentials.	WBC: 38 K/mm^3^ Range (10–38) Neut: 80% WBC returned to normal after amputation.	Initially patient got a total of 4 biopsies without a definitive diagnosis. Patient got excisional biopsy of the mass follow by radiation for a total amount of 6138 rads in 26 cycles over a period of 38 days. He develops ulcerations at the site of treatment which resulted in erosion of the femoral artery with massive hemorrhage; urgent amputation was done.	Amputation resulted in total remission for a period of over 18 years. Patient was alive and well at last follow up.	WBC returned to normal after amputation. No palpable mass seen in the amputated specimen, surgical margins were tumor free, and 2 saphenous lymph nodes were free of metastasis as well. However, positive tumor foci identify during microscopy. Since residual tumor was found in the amputated specimen, these researchers concluded that this malignancy is not completely eliminated by radiotherapy.

Vilanova et al. *Virchows Arch.* (1980) Case report	Sex: Female Age: 71 Presented with left upper quadrant abdominal pain for 3 weeks.	Tumor in the left retroperitoneal space, above the kidney, partially adherent to spleen, and to the larger gastric curvature. Rounded, soft centrally necrotic mass, measuring 10 cm in diameter. No metastatic foci found anywhere.	WBC: 43 K/mm^3^ (range 24–43 K/mm^3^) Neut-30% Baso-35% Eos-22% Meta: 5%	Exploratory laparotomy with biopsy and frozen section; diagnosed it as fibrosarcoma.	Death: on the 7th post-operative day, 7 days after the onset of leukemoid reaction. Immediate cause of death not reported	These researchers concluded that the leukocytic reaction observed was likely secondary to tumor production of granulopoietic growth factor. BM aspiration disclosed granulocytic hyperplasia with depressed erythropoiesis. This researchers concluded that this malignancy 6+follows a rapid, aggressive course and more so in patients with a blood reaction.

Ballestri et al. *European * *journal of ultrasound* (2011) Case report	Sex: Male Age: 65 Presented with intermittent fever for 1 month, and 10 kg weight loss associated with an upper abdominal discomfort/pain and dysphagia.	Tumor located in the retroperitoneum at the level of the right iliac fossa, next to the right iliac vessels and anterior to the psoas muscle. It had a multinodular appearance with regions of hemorrhage and necrosis; measuring 12.5 cm in diameter.	WBC: 24.1 K/mm^3^ (no range reported) Eos: 3.5 K/mm^3^ WBC, constitutional symptoms, and inflammatory markers returned to normal values after surgery.	Surgical removal of the mass via laparotomy followed by chemotherapy and radiation. (no other details specified)	Patient was recurrence free 180 days after diagnosis.	First description of retroperitoneal IMHF assessed with real time ultrasound, frequency-encoded color Doppler and Spectral Doppler, and contrast enhanced ultrasound.

Singh et al. *International journal of urology * (2006) Case report	Sex: Male Age: 58 Presented with left flank pain and fever for 2 months.	First mass was located in the superior pole of the kidney measuring 10 × 12 cm. Second lesion consisted of three masses, the first in the left posterior thoracic cavity (10 × 10 cm), the second in the lesser sac (6 × 6 cm overlying the aorta and inferior vena cava), and the third in the left renal fossa (8 × 8 cm).	WBC: 105 K/mm^3^ (WBC: 70–105/mm^3^ Neut: 90–98% Raised LAP score)	Initial radical nephrectomy showed xanthogranulomatous pyelonephritis without evidence of malignancy. Subsequently patient present with left sided chest pain, fever, and cough. He underwent open drainage of a left lower lung loculated empyema. Analysis of the fluid showed non-specific inflammation. Finally patient present with left flank pain, decrease appetite, and weight lost. Exploratory laparotomy revealed unresectable tumors.	Death within 30 days of diagnosis of IMFH. Patient passed away Approx 30 days after the onset of leukemoid reaction. Immediate cause of death not reported.	This group concluded that this malignancy has a very strong predilection for local recurrence, and distant metastasis. Chemotherapy has been try in few patient with negligent improve in overall survival. Radiotherapy has shown no obvious benefit. In this case the patient had a very poor performance status and could not tolerate any adjuvant chemotherapy. A high index of suspicion is absolutely required for early diagnosis of this rare entity.

Kato et al. *Surgery today * (2002) Case report	Sex: Male Age: 70 Patient presented with a palpable large elastic hard mass in the right upper quadrant of the abdomen. No constitutional symptoms.	Ct-scan of the abdomen demonstrated a well defined mass measuring 12 × 10 cm. It extended from the hepatic hilum to the liver bed, and it appeared to involve the gallbladder. Ct-scan of the abdomen 4 months later showed a lesion 6.5 × 5.5 cm in the ascending colon. Ct-scan of the abdomen 29 months later disclosed another 4 cm mass in the gastric antrum.	WBC: 50.7 K/mm^3^ (range 11–50.7 K/mm^3^) Neut: 88% Leukemoid reaction subsided after surgery.	Initial the mass was excised completely along with the gallbladder and part of the liver. Tumor recurred 4 months later; a 6.5 × 5.5 cm mass was seen in Ct-scan located in the ascending colon. Right hemicolectomy with regional lymph node dissection was done; no evidence of metastasis. Tumor recurred once more 29 months later; a 4 cm mass was detected by Ct-scan in the antrum of the gastric wall. Distal partial gastrectomy with regional lymph node dissection was performed; no evidences of metastasis.	Patient has survived more than 1095 days since he was originally diagnosed and was disease free at last follow up.	Pathology report of initial surgery disclosed a lesion with biomorphic make up: typical IMFH histology and xanthogranulomatous. Thus researchers concluded that the entity known as malignant xanthogranuloma are in fact IMFH. WBC, inflammatory makers (CRP) and serum G-CSF were elevated before surgery. Tumor cells stained diffusely for G-CSF. This article suggested that since a major part of the tumor had xanthogranulomatous histology and only a small portion of it consist of classic IMFH, multiple biopsies must be taken when the diagnosis of IMFH is suspected.

Roques et al. *American cancer society * (1979) Case report	Sex: Female Age: 53 Patient presented with a history of tiredness, backache, ankle edema, anorexia, and weight loss. Patient was afebrile but cachectic with a mass that resembled an enlarged spleen extending 6 cm below the costal margin.	Large (dimensions not specify) soft tissue tumor overlying the left kidney behind the pancreas. This lesion was adherent to left kidney, left adrenal gland, spleen, and pancreas.	WBC: 32.6 K/mm^3^ (range 22.6–32) Neut:88–90% Band N: 9% Mono: 3% WBC count fell to normal levels, but toxic granulations and ALP remained elevated after first surgery. Once tumor relapsed the WBC increased again.	Patient misdiagnosed with chronic neutrophilic leukemia, thus she was treated with Busulphan 4 mg/day. Left upper quadrant mass decreasing in size but Busulphan was discontinued due to pancytopenia. Previous treatment unsuccessful and patient remained symptomatic and she developed fever. Exploratory laparotomy resulted in the resection of a large soft tissue tumor together with the spleen, left adrenal, left kidney and distal third of the pancreas. Five months later, patient developed hepatomegaly and leukocytosis. A subsequent laparotomy revealed widespread peritoneal and hepatic metastases which could not be resected but were biopsied.	Death: Approx 334 days after she first seek medical attention. Patient passed away 334 days after the onset of leukemoid reaction. Immediate cause of death not reported.	Base on a negative Philadelphia chromosome and an increase leukocyte alkaline phosphatase, patient was diagnosed with IMFH associated with leukemoid reaction. The fatal course seen in this case supports the observation that IMFH associated with leukemoid reaction follows an accelerated lethal course. These researches hypothesized that the mechanism responsible for the granulocytic reaction is unlikely to be related to necrosis since this is not a major feature of these tumors. It is probable that IMFH produces a granulopoietic factor.

Takahashi et al. *Pathology, research and practice * (1989) Case report	Sex: Male Age: 77 Patient had a history of recurrent soft tissue tumor in the right scapula that has been surgically removed twice. During his second recurrence he was being closely followed as outpatient, but he fell, bruised his right chest and was admitted to the hospital with severe dyspnea.	On physical exam a non-tender, ill-defined mass was palpated in the right scapular region. Surgical specimen consisted of an elastic, soft, white yellow mass measuring 10 × 7 × 5 cm which was located within the subcutaneous tissue.	WBC: 38 K/mm^3^ (range 22.6–38 K/mm) Neut: 78% Bands N: 8% Blast: 1% Myel: 3% Meta: 2% Fever, increase inflammatory markers, and elevated WBC were elevated when the tumor recurred, and they subsided after the tumor was removed.	Tumor was surgically removed. Originally the patient developed a hen's egg-sized tumor in the right scapula which was excised 10 years before presentation without any further interventions. Five years after the original surgery, tumor recurred and was surgically removed once more.	Death: Occurred 35 days after admission, and the leukemoid reaction begun around the same time. Patient's course complicated with severe respiratory distress secondary to a pneumothorax. Immediate cause of death resulted from cardiac failure.	It is known that the inflammatory infiltrate seen in this tumor occurs with or without necrosis; making the latest an unlikely cause. These researchers demonstrated neutrophil chemotactic activity in tumor extract samples; suggesting that this malignancy can produce this biomolecules.

Algabra et al. *Hystopatho-logy * (1989) Brief Report	Sex: male Age: 67 Patient presented with fever and right epididymis mass. Ultrasound showed a mass extending from the head of the epididymis to the spermatic cord.	Tumor described as a lobulated grey-white mass, measuring 5 × 4.5 × 4 cm that was displacing the vessels and ductus deferens.	WBC 32.5 K/mm^3^ Range (not available) Eos: 7.8% After surgery WBC returned to normal, and the fever subsided.	After radical orchiectomy patient received 10 cycles of chemotherapy (agents not specified) and local radiation with Cobalt 60.	Patient was last since disease free 335 days after surgery.	This is the first case of IMFH of the spermatic cord. This group concluded that the prognosis of spermatic cord IMFH is favorable, with disease free intervals of up to 5 years.

Serke et al. *Oncology * (1986) Case report	Sex: Female Age: 63 Patient presented with progressive hoarseness over 6 weeks. A right 2 × 2 × 2 cm thyroidal mass was palpated. Four week after the first surgery patient presented with a rapid enlarging mass in the right jugulum and hoarseness.	Tumor located in the right thyroid 2 × 2 × 2 cm mass. Tumor (found 4 weeks after surgery) extending from the right thyroid to the aortic arch measuring 3 × 3 × 3 cm. The histopathology of both specimens was exactly the same.	WBC 35 K/mm^3^ (range 6–35) Eos: 40–50% At initial presentation the WBC was normal. It increased after the tumor relapsed.	First surgery was a right sided hemithyroidectomy. Patient refused radiation. Second tumor resected with dimensions as described. After first surgery patient refuse radiation therapy. Patient got a tracheostomy tube after second surgery secondary to an episode of severe shortness of breaths.	Death: 49 days after presentation. Patient died 21 days after the onset of leukocytosis. Immediate cause of death not specified	Eosinophilopoietic factor activity was confirmed in both the serum and IMFH specimen of the patient. These researchers concluded that the tumor was capable of producing growth factors that stimulated the proliferation of eosinophils.

Hurtado-Cordovi et al. Case Report in Medicine (2012) Case report	Sex: Male Age: 60 Patient presented to the general medicine clinic complaining of a rapid growing ulcerative mass in the left arm, just distal to the deltoid muscles. However, patient did not complaint of any significant pain or constitutional symptoms.	Tumor was located in the lateral aspect of the left arm; described as a fungating, necrotic mass measuring 4.4 × 3 × 3 cm. Pre-surgical chest X-ray showed no metastasis.	WBC 109 K/mm^3^ (range 93.6–109) AbsN: 64.5–94.4 K/mm^3^ Abs Bands: 8.6–25.2 K/mm^3^ Abs Mono: 2.1–12 K/mm^3^	Surgery consisted of wide excision of the mass. Specimen's margins were disease free. Patient was evaluated for palliative chemotherapy but due to his critical condition treatment could not be given.	Death occurred Approx 38 days after surgery and 3 days after the onset of leukemoid reaction.	This is the first time that subcutaneous IMFH has been associated with leukemoid reaction. This group concluded that the overproduction of growth factors and cytokines by this malignancy, and their interactions with the tumor's microenvironment seem to be responsible for the aggressive nature of this neoplasm. In addition, they observed that the depth of tumor invasion does not necessarily correlate with metastatic potential.

Liarmakopou-los et al. *Case report in oncology* (2011) Case Report	Sex: Female Age: 65 Patient presented complaining of a painful lumbar mass that develop few days after a fall. On physical exam her temperature was 39°C, and there was palpable, fluctuating, erythematous mass in the lumbar area.	A contrast-enhanced ultrasound showed a heterogeneous mass measuring 9.8 × 8 × 11 cm with a rich vascular supply.	WBC 41.3 K/mm^3^ (range not reported) Neut: 87% (no other differential reported)	Patient was misdiagnosed with paraspinal abscess and underwent 3 unsuccessful drainage attempts which were associated with bleeding and significant morbidity. Biopsy confirmed the diagnosis of IMFH and MRI deemed the tumor inoperable. Neoadjuvant radiotherapy was given and was complicated with excessive bleeding requiring multiple blood transfusions. Patient did not benefit from this treatment.	Death occurred Approx 180 days after patient first seek medical attention. Patient passed away 180 days after the onset of leukocytosis. Immediate cause of death not reported.	Even with the availability of modern imaging, the diagnosis of IMFH can be challenging. This case provides evidences that surgical intervention, which is currently the main treatment for MFH, is not always possible secondary to tumor location and/or patient's brittle clinical condition.

Note: The WBC reported correspond to the highest value observed during the course of the illness, the differential WBC count described illustrates abnormally high parameters only.

Abbreviations: Approx. (approximate), c/o (complaining of), Kg (kilogram), lbs (pounds) IMFH (inflammatory malignant fibrous histiocytoma), BM (bone marrow), LR (leukemoid reaction), IL (interleukin), KGF (keratinocyte growth factor), GSF (granulocyte colony stimulating factor), STC (stem cell growth factor), TGF-beta (transforming growth factor beta), rads (absorbed radiation dose), WBC (white blood cell count), Eos (eosinophils), Baso (basophils), Mono (monocytes), AbsN (absolute neutrophil), Neut (neutrophils), Abs Bands (absolute band neutrophils), Band N (band neutrophils), Abs Mono (absolute monocytes), Blast (blastocytes), Myel (myelocytes), Meta (metamyelocytes), Promy (promyelocytes), LAP (Leukocyte alkaline phosphatase), ESR (erythrocyte sedimentation rate), CRP (C-reactive protein).

**Table 2:** Shows a brief summary of the results.

Total number of studies	14

Total number of patients	16
Males:	12 (75%)
Females:	4 (25%)
Age:	62.6 (range: 47–77)

Tumor location	Retroperitoneal: 8 cases (most common, 50%)
	Abdominal cavity: 2 cases
	Extremities/deep soft tissue: 3 cases
	Superficial: 1 case
	Epididymis: 1 case
	Thyroid: 1 case

Most common complaint at presentation	Combination of mass/pain: 13 patients (81.2%)
	Constitutional symptoms: 7 patients (43.8%), weight lost and fever being the most common.

Number of patients that passed away	12
Numbers of patients alive at last follow up	4
Overall Mean Survival	770 days (range: 14–6570, including the patients that were alive at last follow up)

Time of death after onset of LR/leukocytosis (12 out 16 patients)	92 days (range: 3–334)

## Abbreviations

Approx.: Approximate
c/o: (Complaining of)
Kg: (Kilogram)
lbs: (Pounds)
IMFH: (Inflammatory malignant fibrous histiocytoma)
BM: (Bone marrow)
LR: (Leukemoid reaction)
IL: (Interleukin)
KGF: (Keratinocyte growth factor)
GSF: (Granulocyte colony-stimulating factor)
STC: (Stem cell growth factor)
TGF-beta: (Transforming growth factor beta)
Rads: (Absorbed radiation dose)
WBC: (White blood cell count)
Eos: (Eosinophils)
Baso: (Basophils)
Mono: (Monocytes)
AbsN: (Absolute neutrophil)
Neut: (Neutrophils)
Abs Bands: (Absolute band neutrophils)
Band N: (Band neutrophils)
Abs Mono: (Absolute monocytes)
Blast: (Blastocytes)
Myel: (Myelocytes)
Meta: (Metamyelocytes)
Promy: (Promyelocytes)
LAP: (Leukocyte alkaline phosphatase)
ESR: (Erythrocyte sedimentation rate)
CRP: (C-reactive protein).
